# Exploring the Experiences of Compassion Fatigue Amongst Peer Support Workers in Homelessness Services

**DOI:** 10.1007/s10597-024-01234-1

**Published:** 2024-01-29

**Authors:** Bronwyn Leigh Steenekamp, Stephanie L. Barker

**Affiliations:** School of Psychology, University of Southampton, Southampton, UK

**Keywords:** Homelessness, Peer Support, Compassion Fatigue, Organisational Support, Coping Strategies

## Abstract

Peer support workers have lived experiences of the challenges their clients face. While research has shown peer work can benefit recovery, the negative consequences have not been clearly addressed. This study aimed to explore the experiences of compassion fatigue amongst peer support workers in homelessness services, and the coping strategies used. An explorative qualitative design was adopted. Six peer support workers, in homelessness services, were recruited via snowball sampling. Data was collected using semi-structured interviews and analysed using thematic analysis. Five themes were identified: relentless nature of working in homelessness services, change, making meaning of past experiences, organisational support, and personal coping strategies. There were two novel findings: (1) multiple clients recounting traumatic experiences, and (2) being unfairly blamed for lack of progress, exacerbating compassion fatigue. The findings of this study furthers limited research on compassion fatigue and can be used to develop protocols and practices for organisations that utilise peer support.

## Introduction

Homelessness is a significant and increasing burden in the United Kingdom. The reported number of people experiencing homelessness in England in 2023 was approximately 242,000 (Fitzpatrick et al., 2023), with the expectation that this number will rise 32% by 2026 if no considerable changes are made (Sheikh & Teeman, 2018). Moreover, the number of individuals rough sleeping on a single night in Autumn 2022, was recorded as 3,069 (Department for Levelling Up, Housing & Communities, 2023). This is the highest incidence of rough sleeping recorded since 2017 (Department for Levelling Up, Housing & Communities, 2023). However, the number of hidden individuals experiencing homelessness, including non-visible rough sleepers, squatters, and ‘sofa surfers’ is unknown (Rae & Rees, 2015).

This increase in homelessness is a consequence of a number of factors. The ‘cost of living’ crisis, astronomical rents in the private rented sector, increased mortgage costs, failure to build the social homes required, limited housing benefits and inadequate support or access to services have all contributed to increased homelessness (Fitzpatrick et al., 2023).

Despite the policy initiatives and legislature that exist, most notably the Homeless Reduction Act 2017, many homeless people navigate and exit the system without secured long-term accommodation (Fitzpatrick et al., 2023). Additionally, individuals experiencing entrenched homelessness may never engage with services of their own accord (Rae & Rees, 2015). There are numerous barriers that contribute to this, including lack of money, transport, the individual’s immediate priorities, mental capacity, feeling ignored by society, low self-worth, and poor past experiences (Rae & Rees, 2015). Individuals in stigmatised social positions, including homelessness, often withdraw from social support, which reduces the likelihood of them approaching services (Rea, 2023). Moreover, relationship breakdown is understood as a preceding factor to homelessness, as the individual has no social support to rely on when they are faced with the risk of homelessness (Rea, 2023).

Researchers have indicated a disconnection between the services available to individuals’ experiencing homelessness, and the services that are actually required (Schneider et al., 2019). Alongside the material burdens of homelessness, issues of positive self-image, coping with social isolation, drug and alcohol misuse, violence, communicable diseases, and mental illness can affect homeless individuals (Barker et al., 2018; Schneider et al., 2019). However, service providers frequently focus on concrete needs, such as housing and income, and they may lack the training or capacity to attend to psychological needs.

Studies have demonstrated that some of the most important factors in pulling oneself out of homelessness is positive self-esteem, a strong sense of self-worth, and a capacity for hope (Schneider et al., 2019). In a qualitative study conducted by Rea (2023), friendships with other people experiencing homelessness was discussed by half of the sample (*n* = 20). Within these friendships, homeless individuals were able to receive guidance about accessing services and create a community based on similar shared experiences (Rea, 2023). Furthermore, Schneider et al. (2019) explain that the phenomenon of homelessness is best understood through the words of people who have experienced it, and that to ensure services are not unknowingly silencing homeless individuals further, their voices should be heard. This indicates that peer support is a worthwhile approach to engaging with hard-to-reach populations.

Individuals who work as peer support workers have lived experiences of the challenges their clients face (Barker & Maguire, 2017). Due to shared experiences and personal knowledge, the peer support worker is able to form a unique bond with the client to help foster change. The peers remit is to try and alleviate anything in relation to homelessness, including harm reduction, emotion regulation, substance use, tenancy sustainment, and facilitating engagement to services. The peer is encouraged to utilise their personal experiences of homelessness to guide the relationship, advocate for the individual, and support the individual to seek, and engage with, support from services. Throughout this process, a network is created where peers and clients can both receive support. Therefore, peer support is valuable within homelessness services, as the peer is able to reach those who are often socially excluded (Barker & Maguire, 2017). For the purpose of this study, ‘peer support worker’, ‘peers’ and ‘peer mentor’ are used interchangeably.

Research has shown that the peer support model contributes to reducing hospital admissions, reducing relapses, increasing coping skills, and improving the quality of life for those with mental health issues – dependent on the severity of the mental health issue (Barker & Maguire, 2017). Role modelling, shared experiences, and social support are vital aspects of peer support within homelessness services (Barker & Maguire, 2017). Peer mentors with similar traits are seen as a figure of hope, allowing clients to have someone to measure themselves against. Shared experiences of homelessness, mental illness and addiction, helped to build trust and prosocial relationships that facilitate recovery. Additionally, peers are a source of social support for clients, allowing them to feel like they belong, and help to integrate them into a new community. This can help to develop life skills, increase the clients’ social network, and decrease homeless days (Barker & Maguire, 2017).

Peer support not only benefits the person seeking help but can also have positive effects for the peer (Barker et al., 2018; Mead et al., 2001). Findings have showed that working as a peer provider can improve the individuals management of their mental illnesses and general health, enhance their emotional life and self-concept, give meaning to their life, help to build interpersonal relationships, and lead to career development (Moran et al., 2012). More specifically, within homelessness services, peers described benefitting from helping others, as it allowed them to redefine their experiences and drive positive changes (Barker et al., 2018).

However, there are challenges that accompany working as a peer support worker. Peers need to overcome and have an awareness of their own personal difficulties to fulfil their duties (Barker et al., 2018). Research shows that peer support is most effective when peers are able to acknowledge their limits and have the awareness to refer their clients to professional support when necessary (Barker et al., 2019). Occasionally, peers will encounter situations that trigger past experiences. In these instances, boundaries, controlling one’s emotions in front of clients, and organisational support are vital to prevent relapse or losing compassion (Barker et al., 2018, 2019; Rebeiro Gruhl et al., 2016; Miler et al., 2020). However, due to the flexible nature of being a peer, breaking professional boundaries is sometimes seen as necessary and beneficial (Barker et al., 2018). Therefore, it is vital to understand oneself and the consequences of placing the clients’ needs before their own.

Individuals who work in caring professions must be able to establish trust and build rapport with their clients to work toward goals and develop effective treatment plans (Figley, 2017). This can be a difficult and time-consuming process that requires constant empathy from the professional (Figley, 2017), especially when faced with resistance. If service providers attend to the needs of their clients above their own self-care needs, it can lead to detrimental consequences including the development of vicarious trauma, post-traumatic stress disorder, burnout, and compassion fatigue.

The construct of compassion fatigue has played a vital role in raising awareness of self-care issues. However, compassion fatigue is often mistaken for or combined with vicarious trauma and burnout, making it difficult to understand accurately (Austin et al., 2009). Vicarious trauma occurs when the service provider experiences actual symptoms of trauma caused by constantly hearing about traumatic experiences (Bates, 2018). Whereas, burnout is not necessarily related to client contact and may occur in any occupation where the individual feels overworked, and experiences exhaustion and decreased personal accomplishment (Bates, 2018). Since compassion fatigue has only recently begun to be understood, the phenomenon is less defined and there are no universal, agreed upon symptoms and behavioural markers (Smith, 2019). For the present study, compassion fatigue is understood as the condition of being emotionally drained (Smith, 2019) and losing compassion for clients (Lemieux-Cumberlege & Taylor, 2019) due to the nature of working in homelessness.

Descriptions of compassion fatigue include the feeling of having nothing left to give, distancing oneself from patients and families, feeling as if no real change is happening, feeling drained of all energy and it seeping into all aspects of their lives, reminiscing about how they used to be, and feeling like they no longer meet expectations of themselves (Austin et al., 2009). Individuals may develop a negative attitude toward work, experience less empathy towards clients, lose concentration, and have sleep problems (Bates, 2018; Lemieux-Cumberlege & Taylor, 2019). This shows how compassion fatigue can result in negative consequences for the individual. However, it can also be damaging to the person seeking help, as compassion fatigue can influence the quality of care provided, and the relationship between the support worker and the client (Reyes, 2016).

Understanding how compassion fatigue occurs is key to understanding how to prevent it. There are various risk factors that can influence the development of compassion fatigue. These include less work experience, taking on too much responsibility, setting unrealistic goals, low social support, past trauma, poor self-care, and a lack of job satisfaction (Howell, 2012). Additionally, “shared trauma” may also increase the risk for compassion fatigue, as both the client and worker have experienced similar traumas (Tyson, 2007).

However, not all service providers will experience compassion fatigue. Instead, some experience positive results from their work. Having access to support, availability to process traumatic events, an individual’s ability to cope, psychoeducation, training, evidence-based treatment, lesser caseloads, and use of leisure time and self-care can act as protective factors (Reyes, 2016; Kanter, 2007). This highlights the importance of raising awareness about compassion fatigue and ensuring that organisations provide space for employees to reflect and maintain balance in their life.

Staff working in homelessness services are often exposed to traumatised individuals and traumatic situations (Lemieux-Cumberlege & Taylor, 2019). This can be distressing to both the client and the person providing support (Waegemakers Schiff & Lane, 2019). Additionally, working in homelessness requires staff to be persistent (Smith, 2019), as many people experiencing homelessness have lost trust in the systems intended to help (Morse, 2019). Since this population regularly presents with complex difficulties (Waegemakers Schiff & Lane, 2019), it is unsurprising that compassion fatigue is experienced amongst service providers.

Studies conducted in homelessness services have found a number of influential factors with regards to the development of compassion fatigue, specific to working with the homeless population. The amount of time spent face-to-face with clients, setting appropriate professional boundaries, personal and organisational support, job satisfaction and belief in their work, and the internal coping strategies used by the support worker, all affect the onset of compassion fatigue (Reyes, 2016; Handran, 2015; Howell, 2012; Smith, 2019; Wilkins, 2020). These factors are influential because they affect the amount of time the support worker spends thinking about their cases, how they interpret their experiences, and what mechanisms they use to cope. Experienced positively, these factors can be protective, therefore mitigating the experiences of compassion fatigue (Howell, 2012).

When case managers experience compassion stress, it can develop into compassion fatigue unless there are protective factors (Wilkins, 2020). Literature suggests there is a need for organisations to provide support for all staff working in populations with complex needs. This becomes more important when those helping have personal experiences of homelessness, mental ill health, and/or addictions, like in the case of peer support workers (Barker & Maguire, 2017).

## The Current Study

Peer support workers, working in homelessness services, have lived experience of being homeless and the other complex needs that are often comorbid with homelessness, such as substance misuse and mental illness (Barker & Maguire, 2017). While these experiences allow peers to build meaningful relationships with clients based on shared understanding, it may also mean that they are at greater risk of developing compassion fatigue. Many organisations are utilising co-production and peer support, however there is little research on how to protect peers who are vulnerable. Literature has shown that working as a peer mentor can improve personal recovery. However, the negative experiences of peer support workers are not well researched.

While research available has shown that individuals working within homelessness services commonly experience symptoms consistent with compassion fatigue, it has not addressed the population of peer support workers specifically. This means that homelessness organisations that utilise peer support are reliant on research conducted in other populations to develop training and support protocols. Furthermore, the coping strategies that are used to reduce the experience of compassion fatigue are not researched in this population (Smith, 2019).

Therefore, the current study explored the experiences of compassion fatigue amongst peer support workers within homelessness services through qualitative research. The following objectives were addressed: (1) to explore peer support workers experiences of compassion fatigue and (2) to discover the coping strategies used by peer support workers to cope with stress.

The findings of this research will raise greater awareness of compassion fatigue. The current study may help to develop programs and protocols to reduce experiences of compassion fatigue or to guide supervision sessions for peers in homelessness services. Furthermore, the results may highlight areas for future research, since there is a significant lack of research conducted on compassion fatigue and peer support workers.

## Methodology

### Author Positionality

There are two authors for the current study. The primary author, BS, led the data collection and analysis processes. BS has no experiences of rough sleeping or homelessness but acknowledges their lived experiences of mental illness. The secondary author, SB, did not contribute to data collection given their professional relationships with the sample population and provided feedback and guidance. SB has networks within homelessness services. The authors shared a critical realist stance for the study.

### Research Design

An exploratory qualitative design was chosen due to the scarcity of research available on this topic. This design allowed us to explore the participants’ unique feelings and experiences of compassion fatigue, and the tools used to cope, without preconceived hypotheses, relying on the participants own words (Willig, 2013).

### Participants and Recruitment

Participants were recruited using snowball sampling. SB is affiliated with a homelessness service. Through SB’s network, BS attended two meetings at the organisation to become more familiar with potential participants and build rapport. Ethical approval was given by the University of Southampton (ERGO number: 70269, date: 01/2022) prior to recruitment. Individuals who were 18 years or older, spoke English fluently, and were currently working as a peer in the field of homelessness, were eligible to participate in the study. All the peer support workers have received training on psychological approaches to engaging with clients from the organisation, using a psychologically informed environment framework, delivered by registered psychologists. Data was collected until saturation was achieved. A total of six participants were recruited, the sample consisted of five men and one woman. The participants had varied experience of working as a peer mentor ranging from three months to six years. All of the participants worked at a homelessness organisation in Southeast England. The organisation is a psychologically informed organisation that provides services to people in the homelessness pathway. Peers are recruited to the organisation through advertising with local homeless services and asking about previous service users who have expressed interest in helping or working with people experiencing homelessness. A £10 food voucher was given after the interview was complete.

### Data Collection and Analysis

Data was collected in the form of semi-structured interviews. Interviews were 20 min on average (range 16–35 min). A topic guide consisting of 11 questions, created with the overarching objectives in mind, was followed to ensure that significant responses were received. However, since the nature of semi-structured interviews is not restrictive, BS included additional questions and prompts for further relevant opinions and experiences that arose during the interview (Guest et al., 2012; Smith & Osborn, 2007). Four interviews were conducted face-to-face, at a coffee shop where the peers met weekly for meetings, and two interviews were conducted on Zoom. The interviews were recorded and transcribed verbatim. Informed consent was given before the interview started and the participants were debriefed once the interview had ended.

An inductive thematic analysis was conducted using a critical realist stance. Braun and Clarke’s (2006) six basic steps for conducting a thematic analysis were followed. By focusing on meaning across the data set, we were able to observe and better understand shared or collective experiences and meaning (Braun & Clarke, 2012). The codes were created from small – meaning or phrase – units of analysis to ensure that valuable information was not lost (Roller & Lavrakas, 2015). One hundred and forty-eight codes were created. The codes were organised into five themes, described in the results section.

## Findings

**Fig. 1:**
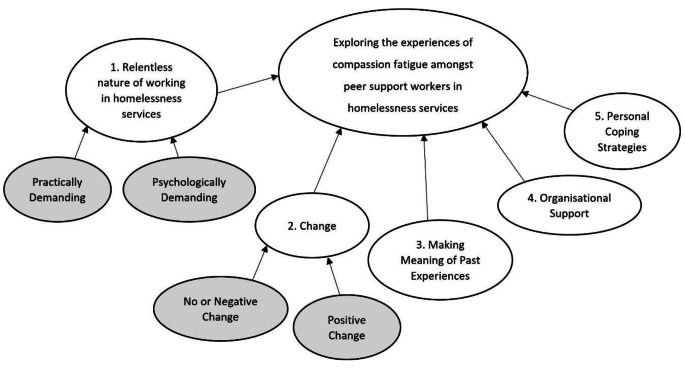
Thematic map

Five themes were identified from the thematic analysis: relentless nature of working in homelessness services; change; making meaning of past experiences; organisational support; and personal coping strategies. These themes and associated subthemes are described below and represented in a thematic map (Fig. 1).

### Relentless Nature of Working in Homelessness Services

This theme encapsulates how demanding peer support work is. It is divided into two sub-themes: practically demanding and psychologically demanding.

#### Practically Demanding

Individuals seeking help for homelessness frequently present with complex needs that extend past the physical difficulties of being homeless (Lemieux-Cumberlege & Taylor, 2019; Smith, 2019). Participants acknowledged the *“multiple needs”* and unpredictability of each client. Furthermore, participants expressed how these needs require persistence from the peer support worker with regards to their knowledge. Participants explained how they are not always sure how to handle new clients’ situations as it “*doesn’t always come straight away”.* Participants addressed how they needed to learn new ways of approaching and interacting with clients to ensure that they are able to communicate clearly with those in difficult situations to facilitate change.

*“I’ve had a few clients that have been quite difficult to cope, to really know what to suggest […] [it’s] learning ways that we can communicate better with service users.” – Richard*.

Participants also explained how some clients were less receptive to how the peer tried to engage with them. In these instances, the participants mentioned needing to *“adapt my working a little bit”* and *“learning new approaches”*. Additionally, participants discussed how it was difficult and time-consuming for them to initially build the relationship with their clients.

*“At first I found it really challenging to get [a client] to kind of engage with me, we kind of didn’t know what was going on. But over the week, she has started to kind of engage more.” – Rory*.

Moreover, participants explained being left to fulfil the duties of other people, even when it is *“not my job”*.

*“I had to tell his ex-partner, or ex-wife actually, that he died […] ‘cause there was no one else in the system could do it. No one. Yeah. The Housing Association said it wasn’t their job. So, I rang.” – Dean*.

#### Psychologically Demanding

Participants discussed the demands that peer work had on their mental health. Participants described witnessing emotionally taxing events. They spoke of how the event affected them at the time, and still does now.

*“I was watching him die. And then he did. So that was really emotional. I still got a little bit of PTSD about that.” – Dean*.

Additionally, participants discussed how it can be *“draining”* to hear from a client experiencing distress. They explained that it is made more difficult when there is *“not a lot you can do either”*. Participants explained how the stories they hear from clients weigh on their mind. They describe how they “*never stop thinking*” about what they have heard. Participants explain that if these instances occur in multiple clients simultaneously, they can experience emotional difficulties like fatigue.

*“There’s some horrendous stories out there and they do affect you. And they do bear on you, […] you do think about them in the evening. You can’t turn them off completely. And they can build up and if you have a few clients that are like that. In one hit, is something you need to bear in mind that they will draw you down, and they will cause you to fatigue.” – Luke*.

Participants discussed how the demands of the work can be overwhelming and fill their thoughts. However, they understood that they “*struggle to do peer mentoring to the level that you want to*” when they are battling with their personal circumstances concurrently. Similarly, participants addressed how clients may “*remind*” them of their past and trigger difficult memories and emotions. They spoke about how these feelings can resurface and cause the peer to struggle again.

*“You remember. That’s the worst part that you remember that you what you felt like when you were at that. And I think that’s where the fatigue comes in, it’s because you remember how you felt, and how low it made you. And it made you lethargic, it made you slow, it made your brain stop working”.– Luke*.

The participants spoke about the weight of responsibility that they felt. They indicated that they would blame themselves if they were not available for their clients when they needed them.

*“If I don’t pick that caller up and they relapse or something happens to them, that’s my fault.” – Jess*.

### Change

This theme focuses on the idea of change, and how a client’s progress or lack thereof can influence the participant. It is divided into two sub-themes: no / negative change and positive change.

#### No Change or Negative Change

Participants describe how difficult it can be when a client is not making any progress, or they are ignoring the help offered to them. They implied that a feeling of defeat develops when the peer has put in effort on multiple occasions and the client “*keeps making the same mistake*”. In these instances, the peers spoke about losing empathy for their clients.

*“When you’re doing the same job with the same people again, you’re seeing rubbish answers constantly or being blanked constantly by people, you know, then you lose that will to help.” – Jess*.

The participants explain that while they are in a position to help the client, they cannot do it for them. The client needs to want to change. However, the participants indicate that it is extremely frustrating for them when a client is not making any changes.

*“You can’t help anybody who don’t want to be helped. That’s a fact. You can’t force nobody, fucking you can take a horse to the water, but you can’t force him to drink. […] But even though you feel like fucking dunking their head in there, do you know what I mean?” – Colin*.

Participants described situations where they worked hard to make a difference for a client, “*we literally did everything*”, but their efforts were unrewarded. They spoke about being unfairly blamed and criticised by the client when the client did not make any progress.

*“And he was saying all sorts of horrible things about me. […] But he couldn’t change his behaviour, and he, it’s all like, blame that on me as an excuse.” – Dean*.

In cases where the client has spoken negatively about the peer, participants discuss ruminating about what the client has said, “*well why did he say that?”*. Participants also spoke about ruminating when they were concerned that they were to blame for a client having a negative outcome, *“did that help his demise?”* Conversely, one participant mentioned that difficult cases can also be *“stimulating”*. However, they implied that there is a solution that would result in a positive change, they just need to figure it out.

#### Positive Change

Participants discussed how seeing a client make positive changes in their lives is rewarding; it can make up for “*all the rubbish clients that you get*”. They spoke about how seeing progress allows them to believe their role as a peer is worthwhile, indicating that the peer is personally invested in the client improving.

*“Gives me strength when I see people get well and see them change. You know, that gives me strength to carry on. Because I know it’s a good thing what we do.” – Richard*.

However, participants acknowledged that you cannot view progression as linear. They explained that they can still view it as a “*success*” if the time between relapses gets progressively longer and the client’s overall quality of life is improving.

*“The first time it might be a couple of hours, it might be a week. The second time might be a month, the third fourth time might be three, four months. So, you know they’re progressing […] but you must remember that there is always going to be where they fall, and they go back down again […] and it’s improving their quality of life.” – Luke*.

Participants described receiving positive feedback from their clients to show that the peer’s help is appreciated, which can help to build the peer’s confidence as it proves they were needed for the change to occur, *“I’m actually making a difference.”*

### Making Meaning of Past Experiences

Participants spoke about how working as a peer support worker is “*a good purpose to be alive*”. They described how in the past they had thought they would not survive, but now they can view their experience as fate, *“like I went through it for a reason.”* Additionally, participants spoke about how helping others in this role gave their life meaning and they felt “*lucky having this opportunity”*. Participants indicated that they were able to feel a sense of achievement and pride from working as a peer.

*“I’ve got something to look forward to, and the fact that very simply, in life, I’m achieving stuff and helping other people, so my life is not meaningless.” – Dean*.

Participants explained that they can now view their past as experience *“almost like a CV”*. Participants were able to accept what they had been through as it taught them meaningful lessons that people who do not have lived experience would be unaware of. They spoke about being needed by the services because of this.

*“Like a normal person would have gone, what do you need to stop drinking? […] But for me, it was like, well, actually, you don’t need to stop drinking right now. What you need is to go down, slowly, […] Where if she was told just stop, would’ve killed her. If she had done that, do you know what I mean. That’s why you need people like me in the services.” – Jess*.

Participants also discussed the career opportunities that working as a peer can bring them. They described how since they started working as a peer they are *“looking forward to like kind of where it could lead”.* Additionally, participants explained how working as a peer allows them to give suggestions about service improvement. They are able to have a voice about how the services run, “*get their point across*” and use their past experiences as an example of what could be done better in the future.

*“Trying to make some changes to the way the services are run. Because they weren’t very helpful for me.” – Richard*.

### Organisational Support

The participants explained that they were comfortable to discuss their personal and work difficulties with their organisation. They indicated that the organisation felt like a safe space where they could express their feelings. They also spoke about being able to rely on everyone within the organisation.

*“On the phone to our assistant psychologist, I burst into tears. But we’re lucky in this organisation We’ve got people who can listen I know I can ring anyone […]The support you get is fantastic” – Dean*.

Furthermore, the participants discussed how they were able to express themselves freely and speak openly about other members of the organisation without worrying that it may not be kept private. Participants spoke about feeling trusted and respected by their organisation. They described being given the freedom to work using methods that suited them without being questioned.

*“I know what I’m doing. Just let me do it. My supervisor is like that, do you know what I mean? That’s why I like working here.” – Jess*.

Participants stated that the organisation offers support to the peers in the form of “*one-on-one*” and “*reflective practice*” sessions. They emphasised the importance of this in maintaining their recovery.

*“Any company that does peer mentoring needs reflective practice and the psychologist to help so it’s not all bottled up, because there’s gonna be people with lived experience, some of that residual. It’s still there. You know, the excess, the propensity to excess.” – Dean*.

In instances where the participants felt they could cope on their own, they still expressed feeling reassured by the organisation, *“it’s nice knowing that there’s somebody there”*. Furthermore, participants discussed receiving support from their colleagues. They explained how they felt able to reach out when they were stuck, and “*bounce off each other*”, which suggests the organisation had fostered an environment that was collaborative. Additionally, participants showed care for their colleagues and spoke about raising concerns about their colleagues’ well-being. Thus, the peers were supported by the organisation, as well as their colleagues.

*“I talk to them first and talk to them about the issue and what’s going on. […] And if I’ve not reassured them, or I feel not confident that I’ve sorted out that they’re okay. Then I will move it up.” – Luke*.

However, one participant mentioned that peer support organisations need to be more proactive in supporting peers. They explained that organisations should pay attention to signs, including changes in work ethic, that a peer may be struggling and act before a problem develops.

*“All these organisations that take peers on whether it’s mental health, addictions, homelessness, whatever. They’re still missing, that they should spend a bit more time gauging their peers. Checking on them, seeing what the consistency of their reports coming back, whether they’re quick and short or whether they’re more informative, if they’re quick and short, then they’re struggling.” – Luke*.

### Personal Coping Strategies

Participants acknowledged the need to care for themselves and ensure that their well-being is being maintained before helping others. They explained that if they put their client’s needs above their own, they may have nothing left for themselves.

*“But above all, put yourself number one, make yourself number one. Because, you know, care about yourself, love yourself. Because otherwise all your love is given to someone else. And none left for yourself, which we need, we need our own self-respect.” – Dean*.

Participants described utilising several coping strategies to reduce stress, including spending time in nature, and exercising as a way to distract themselves from ruminating on their cases. They also described using positive reframing techniques to view difficult situations as opportunities to learn.

*“I have to see the positive and the good in the day. Even when the bad things are happening, especially when there bad things, because they’re the times where I’ve learned the most.” – Richard*.

Participants discussed setting boundaries for themselves and their clients. Participants showed they were aware of their own personal limits. They spoke about declining to work with specific clients or in certain places that might trigger them.

*“So, I was homeless in [city], and I used drugs in [city], then I was working in [city], and you know, clients had this image of me falling around and throwing things and using with a pipe. And in the end I just had to say to my supervisor, look, I can’t do that anymore.” - Jess*.

Similarly, participants spoke about actively setting a clear boundary about their availability and ensuring the relationship remains professional, “*at 5 o’clock I don’t have anything to do with the work phone.”* However, some participants spoke of knowing that this boundary was important but being unable to do it because of a sense of responsibility. Additionally, the participants discussed unhealthy coping tools they used, like immersing themselves in work. However, they expressed awareness that it was *“the wrong thing to do”.*

## Discussion

The current study sought to explore the experiences of compassion fatigue amongst peer support workers in homelessness services and to determine the coping mechanisms that are used. The findings showed that the experiences of compassion fatigue were exacerbated by the psychological demands of working in homelessness services. Furthermore, it was made more difficult if the participants felt that their clients were making no progress, and their efforts were unrewarded, since the participants used their role as a peer to make meaning of their past experiences. However, making meaning of past experiences was also able to act as an indirect coping mechanism for the participants’ recovery. Additionally, organisational support and personal coping strategies were found to reduce the experiences of compassion fatigue.

Interestingly, the participants did not find the practical demands of working in homelessness services as taxing as the psychological demands. Barker et al. (2020) explain that homelessness organisations that utilise peer support go through comprehensive training to ensure that peers are equipped to work in such an environment. This training process is also used to screen potential peer support workers to determine those who are committed to being a peer (Barker et al., 2020). Therefore, peers who have committed to helping may have a good understanding of, and have accepted, the practical demands as the nature of the work. This is supported by Kanter (2007) who explains that social worker distress is frequently caused by unrealistic expectations towards their work. The participants mentioned how working as a peer frequently requires them to learn in order to progress a case. Therefore, a difficult case may be viewed as an opportunity to learn. Thus, it is possible that participants perceive the practical requirements as professional development. This is in contrast with Kanter’s (2007) belief that social workers need to be competent and have all the necessary skills and training already.

Conversely, the psychological demands appeared to have an impact on the experiences of compassion fatigue, in line with the findings from Baker et al. (2007). Participants indicated a feeling of impotence when they witnessed distressing events or were listening to clients in distress on the phone. This is consistent with findings from Waegemakers Schiff and Lane (2019) who reported that working in environments with constant exposure to traumatic situations and traumatised individuals is difficult for the “human psyche”, leading to consequences including compassion fatigue. Furthermore, the participants feelings of fatigue were exacerbated by their own memories. When the participants were reminded of their past, they expressed more difficulties regarding work. This finding is supported by Tyson (2007) and Buttery (2005) who explain that shared trauma can increase the risk of developing compassion fatigue, as the individual is able to closely relate to the client’s experience. Thus, the images and thoughts linked to their trauma may resurface. Similarly, Howell (2012) found that past trauma is an influential risk factor for compassion fatigue, indicating that the peer does not need to have the same experience for their past traumas to exacerbate experiences of compassion fatigue.

Hearing distressing stories was made more draining for the participants when there were multiple clients recounting traumatic situations concurrently. This finding is unique, as there is no known literature that explicitly discusses the number of clients having an impact on compassion fatigue. However, research has shown that spending more time providing direct trauma-related services increases risk of experiencing distress (Handran, 2015), suggesting that more frequent exposure can have an effect on compassion fatigue. Moreover, the participants were further affected when they were dealing with their own personal struggles at the same time, supporting the finding by Figley (2002) that life disruptions can influence compassion fatigue.

The participants’ experiences of compassion fatigue were greater when the clients did not make positive changes. In these instances, the participants reported losing empathy for their clients. This is consistent with literature, Austin et al. (2009) found that nurses experiencing compassion fatigue described giving up when there was no change and the changes they expected never happened. Additionally, the participants feelings of compassion fatigue were exacerbated when they were unfairly blamed for the client’s lack of progress, as it caused the peer to ruminate and doubt themselves. This finding has not been reported previously.

However, when the participants worked with clients that made positive changes they felt rewarded, even more so when the client explicitly expressed gratitude. Reyes (2016) explains that there is a relationship between compassion fatigue and compassion satisfaction. However, the extent to which compassion satisfaction can mitigate compassion fatigue is unknown (Reyes, 2016). In the current study, positive progress seemed to have the ability to combat the participants’ experiences of compassion fatigue, as they expressed that it made up for clients that were unwilling to change or made no progress. Thus, indicating that participants may experience compassion satisfaction when there are positive results for their work.

The participants expressed making meaning of their past experiences and using their experiences as knowledge. Mead et al. (2001) describes how peer support allows peers to make meaning of their experiences by seeing themselves from different perspectives. Therefore, the participant’s ability to find value in their past experiences can act as an indirect coping mechanism for their recovery, as it allows them to see their negative experiences in a positive light. Thus, influencing their abilities to positively reframe experiences. Howell (2012) states that seeing value in one’s work may be a protective factor for compassion fatigue.

Furthermore, the participant’s explained that working as a peer gave them a purpose in life and a reason to live. This thinking places an inordinate amount of pressure upon the peers to have positive outcomes. Therefore, feelings of compassion fatigue may be amplified in cases where there is no progress because the participants have personally invested themselves to such an extent. In cases where the peers do not see the desired outcome, they may feel useless. This is similar to the findings from Austin et al. (2009) who discovered that nurses experiencing compassion fatigue described feeling like they were no longer living up to expectations, which lead to feelings of disengagement, impotence, and hopelessness. Conversely, compassion satisfaction may be heightened when there is progress since client progress can be seen as a personal triumph.

Handran (2015) states that working conditions and the environment of the workplace generate more pressure for caregivers than the trauma survivors they work with. Their study concluded that caregivers who felt supported by their organisation, peers, and supervisors had lower risk of developing burnout, secondary traumatic stress, and compassion fatigue (Handran, 2015). This was demonstrated in the current study. Organisational support was found to be a key factor that influenced compassion fatigue. Participants indicated that the organisation fostered an environment that felt safe, meaning that the peer mentors were able to receive support before something more serious developed. Furthermore, the organisational culture is such that the participants felt comfortable to ask their colleagues for help. Interestingly, this resulted in colleagues feeling comfortable to identify and raise concerns about each other’s well-being if necessary. Therefore, implying that the organisation may have fostered a recovery-oriented environment, where both supervisors and colleagues are mindful of each other’s recovery (Moran et al., 2012).

Barker et al. (2019) explains how trusting the peers’ lived experience, and allowing the peer to work independently, while providing support and structure, is a core element of peer support. Applying this may allow organisations to create a supportive, open environment. The participants indicated that having the organisation’s trust to carry out work freely reinforced their confidence and made them feel respected.

Dearth (2015) explains how individual coping strategies can impact experiences of compassion fatigue, burnout, and compassion satisfaction. The importance of personal coping strategies was highlighted by the participants in the current study. Amongst these coping strategies, positive reframing – where the individual purposefully alters their view of a challenging situation to be more positive (Smith, 2019)–, distraction techniques and boundary-setting were identified by the participants. However, the participants did not specify whether certain strategies were more beneficial.

Setting boundaries has been established as an effective strategy to reduce risk of compassion fatigue (Bates, 2018). Wilkins (2020) found that if boundaries, specifically regarding telephone availability, are not set, it allows for personal and professional life to intertwine, resulting in feeling overwhelmed. Many peers spoke of this boundary, however, not all of them implemented it. Since the participants indicated that they felt the burden of responsibility, it is possible that turning off their phone felt like abandoning their clients without a lifeline.

### Practical Implications

Findings suggest that the emotional demands of working in homelessness services have a great influence over experiences of compassion fatigue amongst peer support workers. Training and support should highlight the importance of self-reflection and self-awareness, to enable peers to determine when they are beginning to feel fatigued, and when they are reacting to situations and client’s differently than before. Furthermore, the findings have indicated that organisational environment is important in reducing compassion fatigue. Therefore, organisations should aim to foster an environment that is open and safe. Opportunities to seek help from a professional psychologist and group reflective practice should be made available. Additionally, the organisation should adopt a relationship with their peers that emphasises trust and respect, by allowing the peers to interact with their clients without being micromanaged.

### Limitations

There are several limitations of the study that should be recognised. The study is fundamentally reliant on the interpretation of the researcher, which is influenced by their identity and epistemological stance. Therefore, the findings may differ if the study were conducted by another researcher. However, this limitation is accounted for by the explicit description of the methods followed, the development of a coding manual (available upon request), and the researcher’s practice of reflexivity. Moreover, the focus of a thematic analysis is on the shared meanings and patterns that are identified in the experiences of the participants. Therefore, a limitation of this method is that some interesting data may be excluded. However, this may also direct the researcher toward new avenues to investigate.

Additionally, SB is affiliated with the homelessness service where the participants work. While the participants were reassured that their information would remain private and confidential, it is not possible to fully anonymise their experiences. Thus, the responses may have been influenced by this relationship. In future, this study should be replicated with a larger sample, to involve multiple homelessness organisations. Additionally, research that attempts to understand the impact of different coping mechanisms on reducing compassion fatigue would be valuable.

## Conclusion

The present study set out to explore experiences of compassion fatigue amongst peer support workers in homelessness services and to discover the coping strategies they use. The literature on this subject is extremely limited, with no previous research focusing on peer support workers in homelessness services. The findings showed that the psychological demands of working in homelessness services were more draining for the participants than the practical demands. The experiences of compassion fatigue were heightened when clients did not make progress towards recovery. Furthermore, the participants used their role as peer support workers to make meaning of their past experiences. Therefore, placing significant importance on their clients making positive changes, as they viewed it as a reflection of their worth. Additionally, the experiences of compassion fatigue were reduced by organisational support and the use of personal coping strategies.

This study furthers the limited research on compassion fatigue and provides clarity on the experiences of the individual. The main contribution of this research is its focus on peer support workers within homelessness services, thereby addressing an important gap in literature. Additionally, a strength of this study is the novel findings: multiple clients recounting traumatic experiences, and being unfairly blamed for lack of progress, exacerbating compassion fatigue. The results of the study suggest demonstratable practices that can be adopted by organisations to ensure the well-being of their peers through training and support.
